# What We Know and Do Not Know About Camouflaging, Impression Management, and Mental Health and Wellbeing in Autistic People

**DOI:** 10.1002/aur.3299

**Published:** 2024-12-24

**Authors:** Valeria Khudiakova, Mishel Alexandrovsky, Wei Ai, Meng‐Chuan Lai

**Affiliations:** ^1^ School of Psychology, College of Life and Environmental Sciences, University of Birmingham Birmingham UK; ^2^ Department of Psychology, Faculty of Arts and Science University of Toronto Toronto Ontario Canada; ^3^ Campbell Family Mental Health Research Institute, Centre for Addiction and Mental Health Toronto Ontario Canada; ^4^ Department of Psychiatry, Temerty Faculty of Medicine University of Toronto Toronto Ontario Canada; ^5^ Department of Psychiatry The Hospital for Sick Children Toronto Ontario Canada; ^6^ Autism Research Centre, Department of Psychiatry University of Cambridge Cambridge UK; ^7^ Department of Psychiatry National Taiwan University Hospital and College of Medicine Taipei Taiwan

**Keywords:** autism, camouflaging, impression management, mental health, stigma, wellbeing

## Abstract

Camouflaging is an impression management strategy employed by some autistic people, widely seen as a response to the pervasive stigma surrounding autism in society. Autistic narratives and lived experiences consistently link camouflaging to anxiety, depression, suicide risks, and autistic burnout. Quantitative research is yet to determine the nature of these relationships, with a significant portion of recent studies providing inconsistent evidence. While camouflaging can be a compelled survival strategy in social environments, it might also contribute to positive outcomes such as securing employment and forming positive social relationships, implicating a complex interrelationship with mental health and wellbeing. We advocate for using a transactional impression management framework to understand camouflaging and wellbeing and address the inconsistencies in research. Through examining the transactions among a person's individual and cognitive characteristics, behavior modification strategies, and the particular social contexts they find themselves in, this framework guides new empirical research directions to delineate the relationships between camouflaging, impression management, mental health, and wellbeing. There is a need to develop multiple measures of camouflaging that delineate the motivations, ability, effortfulness, and perceived effectiveness of camouflaging and examine how a person's social behaviors are perceived in different social environments. Research should also focus on intersectionality, sociocultural influences, and diverse autistic voices to study context‐sensitive camouflaging experiences across the autistic population.

**Summary**

- Some autistic people hide their autistic characteristics from other people, especially when they feel unsafe or not accepted, through a process known as camouflaging.
- However, there is not enough research to conclude whether camouflaging results in poor mental health and wellbeing.
- A lot of the inconsistencies in current research come from the differences in how autistic people's behaviors are viewed, or how they cope with the demands of different social environments.
- By understanding how autistic people with diverse communication, cognitive abilities, and sociocultural backgrounds camouflage in different social situations and how they are viewed by others, we will be able to clarify how camouflaging contributes to different mental health experiences for different people.

Autistic camouflaging is commonly understood as a set of self‐modification strategies aimed at reducing the visibility of autistic traits (Hull et al. 2019). Camouflaging can be conceptualized as a kind of impression management (IM) across human groups: a social coping process of projecting favorable impressions in diverse social environments (Goffman 1959; Leary and Kowalski 1990). However, specific features of camouflaging may be experienced differently and more intensely by autistic individuals (Ai, Cunningham, and Lai 2022), such as its potential psychological impacts. Autistic lived experiences frequently assert that camouflaging leads to anxiety, depression, suicide risks, and burnout (e.g., Bradley et al. 2021; Raymaker et al. 2020). Quantitative research, however, is yet to demonstrate clear causal links between camouflaging and mental health symptoms in autistic people, with some studies finding no significant correlations, and some finding relationships that differ by gender or sex, but with no discernible pattern. No longitudinal studies have been published to date to disambiguate the inconsistencies in existing cross‐sectional investigations (see Khudiakova et al. 2024 for a review).

Leveraging the transactional IM framework (Ai, Cunningham, and Lai 2022), we discuss how gaps in the conceptualization and measurement of camouflaging hinder a comprehensive understanding of camouflaging and mental health in autistic people. We also advocate for participatory research and the thoughtful inclusion of autistic voices and under‐researched groups in future research and consider implications for interventions and novel research avenues.

## 1 What Is Meant by Camouflaging and Transactional Impression Management?

Autism researchers have highlighted the conceptual overlap between camouflaging and established IM constructs (Schneid and Raz 2020; Jorgenson et al. 2020; Hull et al. 2019; Robinson, Hull, and Petrides 2019). For example, camouflaging has been operationalized into three core dimensions of strategies in the Camouflaging Autistic Traits Questionnaire (CAT‐Q; Hull et al. 2019), the most widely used self‐report measure of camouflaging (Hannon, Mandy, and Hull 2023). The three subscales of the CAT‐Q can be contextualized within the broader IM literature (Ai, Cunningham, and Lai 2022). The Compensation subscale corresponds largely to self‐presentation in IM (Jones and Pittman 1982). Both concepts involve deliberate and explicit tactics to appear socially favorable, which are tailored to expectations of particular social situations (e.g., dating, interviews). These active strategies are learned through social exposure, imitation of others, or from studying books or media (Paulhus and Trapnell 2008; Hull et al. 2019). However, learning and enacting these strategies may be more effortful and exhausting for autistic people given that neurotypical social standards are less intuitive to them (Banks et al. 2024). The Masking subscale measures protective strategies that suppress natural autistic behaviors, which corresponds to the concealment of possibly self‐discrediting information across marginalized groups, such as sexual minorities and people with hidden disabilities (Corrigan and Rao 2012; Pachankis, Cochran, and Mays 2015; Pachankis et al. 2020). Lastly, the Assimilation subscale captures one's motivations to blend into social situations and hide any discomfort. This subscale echoes experiences of cultural assimilation (e.g., immigration).

Beyond conceptual overlaps, empirical findings also support the convergence between camouflaging and IM. The three‐factor CAT‐Q structure has been empirically validated in autistic and non‐autistic people (Hull et al. 2019), as well as across the general population in the U.S. (Ai, Cunningham, and Lai 2024a). CAT‐Q scores were also moderately to strongly correlated with scores on the Self‐Presentation Tactics scale in the general population (Ai, Cunningham, and Lai 2024a). Lastly, autistic and non‐autistic people also show similar motivational and mental health correlates of camouflaging (Ai, Cunningham, and Lai 2024a, 2024b). These findings suggest that non‐autistic people also camouflage, likely as part of their IM routines, using similar dimensions of strategies, as driven by similar interpersonal motivations, and while experiencing similar mental health and wellbeing outcomes. However, the strengths of these links among autistic and non‐autistic individuals may be different (e.g., autistic people may experience more intense social pressure and mental health repercussions when camouflaging). It is also important to note that measurement invariance of the CAT‐Q has not been clearly established between autistic and non‐autistic people in non‐English speaking countries, such as France and the Netherlands (Bureau et al. 2024; van der Putten et al. 2023); please see Section 3.2 for a more focused discussion on cross‐cultural camouflaging nuances.

The transactional IM framework conceptually integrates the IM experiences of different groups and different concepts used to describe autistic camouflaging (e.g., self‐presentation, self‐concealment, cultural assimilation, camouflaging, compensation, masking, and adaptive morphing). This framework contextualizes this shared IM experience within the transactions between individuals (including their cognitive and information processing characteristics) and their social environments (Ai, Cunningham, and Lai 2022), thereby addressing how IM consequences, including changes in mental health and wellbeing, manifest in diverse groups across the general population. For example, whereas individuals with higher autistic traits report poorer mental health at elevated levels of camouflaging, those with higher ADHD traits report more positive mental health (Ai, Cunningham, and Lai 2024b). These group differences suggest the need to consider transactional mechanisms in how dimensional individual traits, such as neurodivergent characteristics, interact with social demands. In the following section, we outline how autistic camouflaging, mental health, and wellbeing can be understood through the transactional IM framework.

## 2 A Transactional Impression Management Approach to Autistic Camouflaging, Mental Health, and Wellbeing

The transactional IM framework leverages existing IM literature to pinpoint potential mechanisms in how camouflaging impacts mental health and wellbeing. The key prediction of this framework is that camouflaging, theoretically grounded in IM, may be employed and experienced differently depending on (1) individual traits and cognitive abilities; (2) sociocontextual demands; and (3) individual‐contextual transactions (Ai, Cunningham, and Lai 2022).

First, the “effectiveness” of camouflaging in reducing the perceptibility of one's autistic traits may differ depending on the individuals' cognitive capacities, which may then modulate mental health and wellbeing outcomes. Engaging in and maintaining camouflaging requires social and self‐awareness, executive functioning, and perspective taking capacities (Ai, Cunningham, and Lai 2022). Having fewer executive functioning difficulties has been linked to greater camouflaging in autistic people (Livingston et al. 2018; Hull et al. 2021; Lai et al. 2017). Similarly, in the general population, IM depletes cognitive resources and requires monitoring shifts in social environments, inhibiting and generating behaviors, and inferring others' intentions (Baumeister and Vohs 2012; Critcher and Ferguson 2013). Those with higher cognitive abilities in these domains might find camouflaging to be more “effective,” tailored to particular social contexts and relatively less effortful. Hence, higher cognitive capacities could be protective factors in reducing some adverse outcomes of camouflaging. Meanwhile, those with more cognitive difficulties may strive to “blend in” but struggle to do so despite facing strong social pressures. This can lead to more adverse and taxing experiences. Similarly, camouflaging may have more adverse outcomes in those with greater autistic traits. Empirical data show that individuals with higher autistic traits and higher camouflaging report greater anxiety, depression, and fatigue and reduced mental wellbeing (Ai, Cunningham, and Lai 2024b). More significant autistic traits may entail additional difficulties in reading social cues in neurotypical contexts, possibly making camouflaging more frequently or intensely required across various social settings, more difficult to process, and more taxing to implement, thus contributing to adverse mental health and wellbeing outcomes.

Second, the outcomes of IM and the motivations for it may depend on its social context. Social contexts further vary in their demands, durations, and audience sizes and makeup. For example, Dean, Harwood, and Kasari (2017) observed that the social dynamics of girls' groups in the playground allowed autistic girls to blend in more readily, in contrast with boys' groups, which highlighted the interactive differences of autistic boys. Within the CAT‐Q, the motivation to camouflage as captured by the Assimilation subscale is more consistently linked to adverse mental health and wellbeing outcomes than the other two subscales measuring specific behaviors (e.g., scripting conversations; Khudiakova et al. 2024). This may be because the Assimilation subscale additionally taps into the discomfort, inauthenticity, and sense of compelled action that autistic people feel in neurotypical social contexts (Belek 2023). However, recent psychometric research has questioned the extent to which the Assimilation subscale can distinguish between camouflaging, social autistic traits, and social anxiety (McKinnon et al. 2024). Nonetheless, the degree to which camouflaging is voluntary versus a form of forced social modification (Ai, Cunningham, and Lai 2022) may be key to disambiguating its mental health and wellbeing effects. In dominant social groups, camouflaging and IM may be employed instrumentally to enhance one's social standing, whereas marginalized groups, including autistic individuals, are often compelled to camouflage as a means of survival and avoiding harm (Pearson and Rose 2021). Indeed, a path‐analytical study by Zhuang et al. (2024) found that autistic people who were more vulnerable to negative life events were more likely to camouflage. This coerced nature of autistic camouflaging may add an additional layer of burden beyond fatigue in neurotypical‐dominant social contexts.

Third, camouflaging may be understood as a transaction between a person's IM “performance” and a particular “stage”. This is in line with the developmental contextualism view, which entails that individual traits and social environments reciprocally influence each other throughout development (Thomas and Chess 1981; Rauthmann 2021). In IM research, the degree of stigmatization is associated with one's identity, the specific social demands, and the accessibility of sociocultural conventions, all of which may affect one's engagement in IM (Goffman 1959; Leary and Kowalski 1990). For instance, in people holding stigmatized but not immediately apparent social identities, believing their identities to be more concealable correlates with reduced anxiety around intergroup interactions (Le Forestier et al. 2020). This might similarly apply to autistic people. Autism‐specific IM experiences may emerge from reciprocal processes between individual traits (e.g., sociodemographic and dispositional traits), cognitive characteristics (e.g., executive functioning, metacognitive awareness, prior social beliefs and motivations), and the demands of a particular social context (e.g., the need to hide autism‐related stigmatized traits). This transactionality is supported by emerging qualitative research. For many autistic adults, camouflaging involves understanding the expectations of social contexts and then deciding to either meet or subvert them (Khudiakova 2024). Hence, camouflaging can be viewed as an autism‐specific form of IM, and the deeper understanding of which should come from the concurrent consideration of the individual traits and cognition, contexts, and the interactions between them.

In the following sections, we underscore how the transactional IM framework is vital in identifying currently uncaptured and unmeasured aspects of camouflaging, how they may impact mental health, and potential directions for future intervention efforts. The transactional IM framework is thus not to serve as an analogy of camouflaging, but a foundation for contextualizing it. We emphasize what we can learn from the shared context‐dependent and transactional foundations of camouflaging and IM to enhance our understanding of autistic camouflaging, mental health, and wellbeing.

## 3 What Is Missing From Autistic Camouflaging and Mental Health Research

These individual, contextual, and transactional aspects of camouflaging under the transactional IM framework should be carefully delineated, as they may show different associations with mental health and wellbeing. So far, research has not sufficiently included individuals with diverse needs, been conducted across sociocultural contexts (see Section 3.2), and considered individual‐context transactions. Camouflaging is multifaceted, so its measurement should extend beyond self‐perceived camouflaging frequency and intent (as measured by the CAT‐Q). More targeted measurement tools for different aspects of camouflaging such as ability, effectiveness, effortfulness, and the associated contextual factors are needed.

### 3.1 The Need for Measurement Precision

The diversity of current camouflaging measures poses challenges and opportunities. Current measures were not initially developed to capture the conceptual complexity of camouflaging delineated by the transactional IM framework (Hannon, Mandy, and Hull 2023; Williams 2022), which limits our ability to draw conclusions about the relationship between camouflaging and mental health using available measures to date. These measures include introspection (e.g., self‐report questionnaires), observation (e.g., behavioral coding or informant‐report questionnaires), and the discrepancy between observable behaviors and one's “internal autistic status” (Hannon, Mandy, and Hull 2023). Self‐report data capture perceived camouflaging intent and self‐perceived behaviors (Hull et al. 2019), while behavioral observations can demonstrate the effectiveness of camouflaging across more naturalistic settings with third‐party social evaluations. The discrepancy approach could be useful in clinical settings when identifying autistic people whose behavior might not outwardly seem atypical but who still experience social difficulties. Although these measures are all treated as representing camouflaging, the facets measured are likely different (although related). This is supported by Hannon, Mandy, and Hull (2023), who found that the discrepancy measure was only weakly correlated with the CAT‐Q. Despite multiple available measures, current findings are over‐reliant on self‐report data, which are limited to capturing consciously controlled camouflaging and its impacts (Hannon, Mandy, and Hull 2023; Cook et al. 2021).

Camouflaging strategies can be learned and enacted automatically or deliberately (Howe et al. 2023; Cook et al. 2021; Perry et al. 2021). Autistic lived experiences suggest that consciously controlled camouflaging can become ingrained and unconscious after accumulated experiences (Lawson 2020). Whether automatic camouflaging might become conscious over time, such as through self‐reflection or social feedback, requires further investigation. Automatic and controlled camouflaging could contribute to different behavioral presentations and mental health outcomes, but empirical studies are needed to unpuzzle these contributions. Automatic camouflaging may have initially emerged as an intuitive defense mechanism learned implicitly due to social pressures (e.g., harassment, exclusion, harm), as many autistic people are unaware of having been observing and imitating others (Lawson 2020). It is challenging to articulate, understand, and measure these implicit camouflaging tendencies and associated mental health and wellbeing risks. Being unaware of one's camouflaging could mean that these efforts do not elicit the same sense of discordance with one's “true self” as controlled camouflaging often does (Evans, Krumrei‐Mancuso, and Rouse 2023), although the psychological toll may persist. Conversely, it is also possible that consciously controlled camouflaging allows for a greater sense of control during IM, which diminishes negative mental health and wellbeing consequences such as persistent social fear and anxiety. Understanding how automatic and controlled camouflaging strategies coexist and co‐evolve across development in different social contexts is crucial for assessing their impacts on mental health and wellbeing.

The transactional IM framework helps advance measures that delineate the “effortfulness,” “effectiveness,” “ability”, and “motivations” of camouflaging, which will enable researchers to assess why some autistic people camouflage successfully, others strive but struggle, and yet others remain unmotivated despite social pressure. We anticipate the development of new complementary measures of camouflaging—as a kind of transactional IM—that centre around the contextual differences in how a person acts and how their behaviors are perceived. Drawing from Williams' (2022) “experimental discrepancy” conceptualization, we propose comparing individuals' camouflaging experiences across experimentally manipulated social scenarios. The social stimuli involved should capture the dynamics and contingencies in naturalistic social interactions and vary in the extent of social demands, stakes, interaction partners, and contexts. Scenarios could range across meeting strangers, job interviews, romantic dates, cocktail parties, and family gathering, among others. Autistic participants can report on the types of adopted strategies, aspects of themselves that were monitored, efforts involved, and the physical and mental costs of these efforts. Recorded interactions can be shown to future participants across neurotypes to capture observer social “appropriateness” and favorability evaluations. In the end, operationalizing camouflaging using both internal and external metrics across social scenarios could clarify the transactional mechanisms that determine camouflaging effectiveness, automatic vs. controlled aspects (e.g., what was observed vs. reported), and the associated mental health outcomes.

### 3.2 The Need for Intersectional and Sociocultural Research

The transactional IM framework suggests that the effects of camouflaging on mental health are likely to vary by sociocultural context (Ai, Cunningham, and Lai 2022). Research integrating intersectional perspectives is therefore essential to properly delineate diverse context‐dependent camouflaging experiences in autistic people and why they occur (Radulski 2022).

Camouflaging is essentially an IM effort to fit into one's sociocultural surroundings and should thus show cross‐cultural nuances. For instance, instead of the commonly found three‐factor structure of the CAT‐Q in Western autistic and non‐autistic people (Hull et al. 2019; Ai, Cunningham, and Lai 2024a), a two‐factor structure was found in Taiwanese autistic and non‐autistic adolescents (Liu et al. 2024), and the CAT‐Q showed limited factor validity in Japanese autistic and non‐autistic adults and thus had to be substantially adapted (Hongo et al. 2024). It may even be possible that camouflaging shows greater variation between autistic people from different sociocultural backgrounds than between autistic and non‐autistic people from the same background, although the cross‐cultural evidence for which is scarce. As such, it is vital to study camouflaging beyond those in English‐speaking countries or “WEIRD” (White, Educated, Industrialized, Rich, and Democratic) populations (Henrich, Heine, and Norenzayan 2010). Different cultural, linguistic, and attitudinal perceptions of autism may necessitate divergent camouflaging tactics and color the perceptions of autistic people (e.g., Liu et al. 2024; Tamura et al. 2024; van der Putten et al. 2023). The development of more culturally and ecologically valid measures would further help clarify the relationship between camouflaging, mental health and wellbeing cross‐culturally.

Additionally, current findings about camouflaging are not generalizable to autistic individuals who are minimally/non‐speaking or have intellectual disabilities (Cook et al. 2021; Zhuang et al. 2023). Their experiences of stigma and interpersonal trauma might differ from those with fewer difficulties in verbal communication or social reasoning (Harris et al. 2008; Jacobs, Simon, and Nader‐Grosbois 2020), especially in the foundational supporting cognitive abilities of camouflaging/IM, such as executive functioning and perspective taking. This could affect their interpretation of and responses to social conventions and pressures. Although direct data from these groups are scarce, it is plausible that they are the least “effective” in camouflaging and thus more vulnerable to threats of harm and stigmatization. Many camouflaging strategies, such as mimicking speech or memorizing conversation scripts, may be impractical for minimally/non‐speaking autistic people. For this group, camouflaging intent and efforts could be qualitatively different or unrecognized, and the associated responses might differentially influence mental health and wellbeing.

It is essential to recognize other intersectional impacts in camouflaging. In dominant social groups, IM may be employed strategically and voluntarily (Leary and Kowalski 1990). However, minoritized individuals may resort to IM more defensively, aiming primarily to conceal stigmatized traits (e.g., among sexual minorities; Larson and Chastain 1990; Pachankis et al. 2020), representing intentions similar to the involuntary and coerced social adaptation experienced by autistic individuals (Lawson 2020). The experiences of camouflaging in autistic individuals with additional stigmatized identities, such as racialized and minority gender or sexual identities, may be unique but remain under‐explored (Malone et al. 2022; McQuaid, Lee, and Wallace 2022; McQuaid et al. 2023). Simultaneously managing multiple stigmatized identities can intensify the challenges of camouflaging, leading to greater mental health strain. A merging of neurodevelopmental and social psychological research is therefore necessary to offer intersectional analyses for how camouflaging impacts mental health and wellbeing.

### 3.3 The Complex Relationships Between Autistic Camouflaging and Mental Health/Wellbeing

Possible non‐linear relationships between camouflaging and mental health/wellbeing may exist (Hull et al. 2021; Ai, Cunningham, and Lai 2024b). For instance, initial outcomes of camouflaging could become its drivers, thus perpetuating and exacerbating the same outcomes through a feed‐forward mechanism. This possibility is highlighted by the dilemma of camouflaging. For example, although camouflaging may allow autistic individuals to build new friendships, these friendships may come with greater feelings of social disconnection due to one's awareness of their inauthentic expressions (e.g., the feeling that one is not fully understood or accepted). In another instance, camouflaging may allow autistic individuals to hide from stigmatization, but the threat of societal stigma persists and remains unchallenged. In both cases, camouflaging is perpetuated and its mental health and wellbeing costs likely exacerbate. The transactional IM framework (Ai, Cunningham, and Lai 2022) illustrates such a hypothetical feedback loop, which may guide future experimental, interventional, and longitudinal studies to assess the causal and developmental relationships between camouflaging and mental health/wellbeing.

There is also a need to understand the extent of the conceptual overlap between camouflaging and behaviors related to social differences of other origins, such as neurodivergence in learning or specific mental health experiences, particularly social anxiety. For example, it has been proposed that camouflaging may represent a behavioral response associated with social anxiety (Lei et al. 2023), which might explain the high correlations between camouflaging and social anxiety symptoms often observed in questionnaire‐based research.

Camouflaging may indirectly lead to increased mental health and wellbeing challenges, such as self‐blame, depression, and anxiety; these problems can be exacerbated by dismissed formal and timely diagnosis and support, which are, in turn, worsened by camouflaging (Hull et al. 2017; Bradley et al. 2021; Lawson 2020; Milner et al. 2022). There may also be common causal mechanisms between camouflaging and mental health/wellbeing that may explain their associations; for example, elevated social awareness or loneliness may increase both camouflaging and mental health/wellbeing difficulties. Thus, longitudinal research is needed to clarify these complex relationships. Such studies would establish temporal precedence and track how camouflaging experiences and outcomes co‐evolve in response to changing social demands, especially in key life stages and transitions (e.g., adolescence to young adulthood). This design could help identify socio‐contextual and developmental factors that precipitate camouflaging, how camouflaging repertoires develop among intersectional groups with varying abilities and intents to camouflage, and how these factors influence mental health and wellbeing.

## 4 Future Avenues for Research and Services

If camouflaging is identified to be causally aggravating certain mental health difficulties, interventions that may inadvertently instil such strategies should be refined. For example, fostering community connectedness may prove particularly beneficial (Petty et al. 2023) in mitigating the adverse effects of camouflaging on mental health and wellbeing. Instead of emphasizing behavioral modifications and treatment outcomes based on neurotypical standards (e.g., number of friendships, social initiations), interventions should prioritize outcomes that genuinely improve the wellbeing of autistic people. This can include creating individual‐tailored opportunities for autistic people to build meaningful and long‐lasting friendships, or learn strategies to find accommodating and meaningful social networks and build confidence and positive self‐growth. Accordingly, current social skills‐building efforts should place these individual‐tailored goals at the centre of intervention and, critically, should strive to support the autistic individual *and* change their social environment and demands at the same time.

It is also crucial to differentiate between relatively beneficial and damaging camouflaging strategies to develop balanced interventions. Critically, the benefits and drawbacks of strategies could vary by person and context. Therefore, it is important to approach the recommendation to “unmask” with caution. Reducing camouflaging may not always be feasible or lead to better mental health and wellbeing. Positive outcomes such as securing employment and social relationships highlight the adaptive functions of IM in both autistic and non‐autistic people (Zhuang et al. 2023; Alaghband‐Rad, Hajikarim‐Hamedani, and Motamed 2023; Field et al. 2024). Additionally, removing camouflaging could expose autistic individuals to heightened anti‐autism discrimination prevalent in social spaces. The stakes of not camouflaging are especially heightened for racialized groups (Radulski 2022). A context‐informed, transactional IM analysis would contribute to the development of new support approaches that incorporate a range of social coping strategies beyond modifying the autistic individual, aiming also at changing neurotypical social spaces and community bridging (Kim et al. 2024). These programs should be tailored to the needs of each autistic individual considering their intersecting identities and sociocultural backgrounds. A complementary approach is to tackle internalized stigma, thereby alleviating both its direct adverse effects on mental health/wellbeing and indirect impact through camouflaging (Ai, Cunningham, and Lai 2024b). In any case, advocacy efforts should be supported to prioritize cultural sensitivity and systemic change to create environments conducive to the wellbeing of all autistic individuals.

Autistic voices must be centred in camouflaging research and any relevant interventions, particularly from those with diverse intellectual and communication abilities (Tesfaye et al. 2023). Participatory research ensures the inclusion of diverse perspectives in studying autistic camouflaging, mental health, and wellbeing to avoid the perpetuation of neurotypical hegemony (Chown et al. 2017). Collaborating with autistic people facilitates a deeper understanding of lived experiences, which is integral for understanding camouflaging and the fine‐grained factors influencing autistic people's interpersonal interactions across sociocultural contexts. Integrating these insights when formulating hypotheses, designing experiments and/or analytical approaches, interpreting data, and developing theories in studies on camouflaging, mental health and wellbeing enhances the validity and generalizability of findings.

## 5 Conclusions

We advocate for (1) the adoption of the transactional IM framework to delineate the motivations, ability, effortfulness, and effectiveness of camouflaging in a transactional manner; (2) the intentional development and use of different context‐sensitive measures to capture camouflaging; and (3) the consideration of intersectionality through the interplay between individual, sociocultural, and developmental factors, to properly understand the mechanistic relationships between camouflaging, mental health and wellbeing in autistic people. Through enhancing conceptual and methodological clarity and meaningfully including autistic people in its full diversity in research, a more comprehensive and ecologically valid understanding of camouflaging and mental health and wellbeing can be fostered. Ultimately, these efforts will help navigate and define optimal social coping and create individualized support to enhance the mental health and wellbeing of autistic people.

## Ethics Statement

The authors have nothing to report.

## Conflicts of Interest

The authors declare no conflicts of interest.

## Data Availability

Data sharing is not applicable to this article as no new data were created or analyzed in this study.
